# Mild cognitive impairment, poor episodic memory, and late-life depression are associated with cerebral cortical thinning and increased white matter hyperintensities

**DOI:** 10.3389/fnagi.2014.00306

**Published:** 2014-11-07

**Authors:** Motonobu Fujishima, Norihide Maikusa, Kei Nakamura, Masahiro Nakatsuka, Hiroshi Matsuda, Kenichi Meguro

**Affiliations:** ^1^Department of Nuclear Medicine, Saitama Medical University International Medical CenterHidaka, Japan; ^2^Integrative Brain Imaging Center, National Center of Neurology and Psychiatry (NCNP), KodairaTokyo, Japan; ^3^Division of Geriatric Behavioral Neurology, Cyclotron and Radioisotope Center, Tohoku UniversitySendai, Japan

**Keywords:** mild cognitive impairment, episodic memory, late-life depression, cortical thickness, white matter hyperintensity

## Abstract

In various independent studies to date, cerebral cortical thickness and white matter hyperintensity (WMH) volume have been associated with episodic memory, depression, and mild cognitive impairment (MCI). The aim of this study was to uncover variations in cortical thickness and WMH volume in association with episodic memory, depressive state, and the presence of MCI simultaneously in a single study population. The participants were 186 individuals with MCI (clinical dementia rating [CDR] of 0.5) and 136 healthy elderly controls (HCs; CDR of 0) drawn from two community-based cohort studies in northern Japan. We computed cerebral cortical thickness and WMH volume by using MR scans and statistically analyzed differences in these indices between HCs and MCI participants. We also assessed the associations of these indices with memory performance and depressive state in participants with MCI. Compared with HCs, MCI participants exhibited thinner cortices in the temporal and inferior parietal lobes and greater WMH volumes in the corona radiata and semioval center. In MCI participants, poor episodic memory was associated with thinner cortices in the left entorhinal region and increased WMH volume in the posterior periventricular regions. Compared with non-depressed MCI participants, depressed MCI participants showed reduced cortical thickness in the anterior medial temporal lobe and gyrus adjacent to the amygdala bilaterally, as well as greater WMH volume as a percentage of the total intracranial volume (WMHr). A higher WMHr was associated with cortical thinning in the frontal, temporal, and parietal regions in MCI participants. These results demonstrate that episodic memory and depression are associated with both cortical thickness and WMH volume in MCI participants. Additional longitudinal studies are needed to clarify the dynamic associations and interactions among these indices.

## Introduction

Mild cognitive impairment (MCI) is a heterogeneous clinical condition that may precede Alzheimer disease (AD) as well as vascular and other dementias (Meyer et al., 2002). In patients with MCI, memory is impaired whereas other cognitive functions are relatively spared (Petersen et al., 1999). With regard to features of the brain observed with structural MRI, compared with healthy elderly controls (HCs), MCI patients tend to show decreased cortical thickness in the temporal lobe, reduced hippocampal volume (Liu et al., 2011), and increased white matter hyperintensity (WMH) volume in the cerebrum (Smith et al., 2011; Iorio et al., 2013; Yates et al., 2014). More recently, an association between decreased cerebral cortical thickness and increased WMH volume has been reported (Seo et al., 2012). Furthermore, increased WMH volume is thought to be related to normal aging and hypertension as well as decreased cognitive functions (Smith et al., 2011; Rostrup et al., 2012; Birdsill et al., 2014).

Patients with MCI are at increased risk of progression to dementia (Richard et al., 2013), of which manifestations of depression could be regarded as early symptoms (Panza et al., 2010). In the context of associations between depressive symptoms and structural changes in the brain, reductions in amygdalar volume, hippocampal volume, or both have been reported in elderly patients with depression (Egger et al., 2008; Burke et al., 2011). Associations between WMHs and affective disorders in elderly populations have also been elucidated. Herrmann et al. (2008) reviewed the literature and reported that WMHs are also observed more frequently in elderly patients with depression than in controls. In the study by Disabato et al. (2014), patients with late-onset late-life depression had increased WMHs and thinner cortices in the left anterior cingulate relative to those with early-onset depression. Kieseppä et al. (2014) showed that only middle-aged patients with bipolar disorder type I were at increased risk of deep WMHs, which in turn were independently associated with deficits in visual attention. Serafini et al. (2010) reported that MRI findings of deep WMHs could be a useful biological predictor of severity in patients with late-onset late-life bipolar disorder type II.

As described above, various clinical features in MCI are mutually interrelated. However, few studies have investigated the relationships among these features in a single study population. Therefore, in the present study, we explored the following questions. (1) Do MCI patients have thinner cerebral cortices (especially in the temporal lobe) and higher WMH volume than do HCs? (2) Does their deteriorated cognitive function, including problems with episodic memory, reflect thinner cortices, increased volume and spatial distribution of WMH in the brain, or both? (3) Compared with non-depressed MCI patients, do depressed participants have thinner cortices, higher WMH volumes, or both, and if so in what regions? (4) Is increased WMH volume associated with cerebral cortical atrophy and its spatial distribution? To address these issues by novel, automated, and quantitative methods, we analyzed the structural MRI scans of MCI and HC participants drawn from two community cohorts: the Osaki-Tajiri (Meguro et al., 2002) and Kurihara (Meguro et al., 2012) projects.

## Materials and methods

### The osaki-tajiri and kurihara projects

Participants were drawn from the two aforementioned community-based cohort studies. The Tajiri project was undertaken to study preventive strategies against stroke, dementia, and bed-confinement with a target population of people aged over 65 in old Tajiri, northern Japan from 1988 (Meguro et al., 2002). The project was renamed the Osaki-Tajiri project in 2005 and recruited 1654 participants. The Kurihara project, whose aims were the same as those of the Osaki-Tajiri project, recruited 590 participants aged over 75 in Kurihara, located to the north of Osaki, starting in 2008 (Meguro et al., 2012). The ethical committees of the Tajiri SKIP Center, Tohoku University Graduate School of Medicine, the Kurihara Central Hospital, and the National Center of Neurology and Psychiatry approved the data collection procedures and data analysis. Written informed consent was obtained from all participants. In the event that the participant was unable to express agreement, a family member signed the informed consent form on behalf of the participant.

### Participants

**Figure 1:**
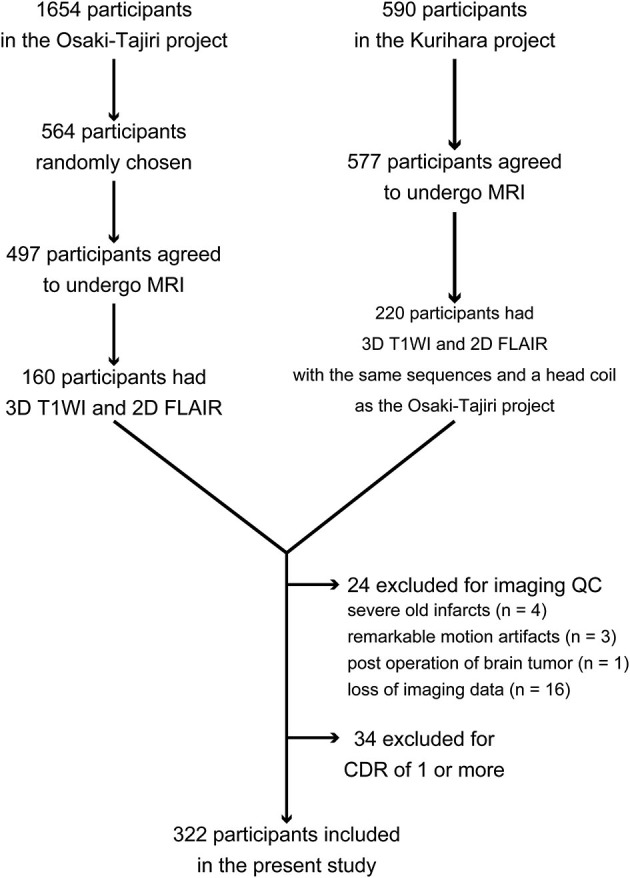
**Flow diagram for inclusion and exclusion of participants in this study**. Abbreviation: QC = quality control.

To perform quantitative analyses on structural MRI scans of the brain to compute cerebral cortical thickness and WMH labeling, the present study required a pair of high-quality images comprising a three-dimensional (3D) T1-weighted image and a two-dimensional (2D) fluid-attenuated inversion-recovery (FLAIR) image. Although 497 participants underwent brain MRI in the Osaki-Tajiri project, only 160 were examined with both 3D T1-weighted imaging and FLAIR imaging. In the Kurihara project, 220 of the 577 participants had both a 3D T1-weighted image and a FLAIR image scanned with the same sequences and head coil as in the Osaki-Tajiri project. Then, through quality control of the imaging data, participants were excluded if they had old infarctions, except for 1 or 2 lacunar infarcts (*n* = 4); remarkable motion artifacts (*n* = 3); post-surgical operation of a brain tumor (*n* = 1); or imaging data loss (*n* = 16). Furthermore, participants with a Clinical Dementia Rating (CDR) of 1 or more were excluded (*n* = 34). Eventually, 322 participants in total (127 from the Osaki-Tajiri project and 195 from the Kurihara project) were included in this study. Clinical demographic data and a flow diagram of the current study are shown in Table 1 and Figure 1, respectively.

**Table 1:** **Demographics of the participants (*n* = 322)**.

	HC (CDR of 0)	MCI (CDR of 0.5)	*p*-value
Number	136	186	
Age, y	78 (76–81)*^b^*	80 (76–83)*^b^*	<0.005
Gender, M/F	55/81	84/102	0.40
Education, y	9 (8–11)*^a^*	8 (8–9)*^a^*	0.01
MMSE	26 (24–27)*^c^*	23 (21–25)*^c^*	<0.001
WMS-R LMII	9 (5–13)*^c^*	4 (1–10)*^c^*	<0.001
GDS-15	3.5 (2–6)	4 (2–7)	0.07
WMHr, %	1.03 (0.54–1.82)*^c^*	1.76 (0.87–3.06)*^c^*	<0.001

*Data are presented as numbers or medians (interquartile ranges). Abbreviations: CDR = Clinical Dementia Rating; F = female; HC = healthy elderly control; GDS-15 = 15-item Geriatric Depression Scale; M = male; MCI = mild cognitive impairment; WMHr = cerebral white matter hyperintensity volume as a percentage of the total intracranial volume; WMS-R LMII = Wechsler Memory Scale-Revised Logical Memory II. ^a^*p* < 0.05, ^b^*p* < 0.005, ^c^*p* < 0.001*.

### Clinical and neuropsychological assessment

All participants underwent clinical and neuropsychological testing. This involved medical interviews with the participants themselves or their family members. Furthermore, we assessed each participant on the following four neuropsychological scales. (1) We used the Japanese version (Otoyama et al., 2000) of the CDR scale (Hughes et al., 1982) to rate each participant’s severity of dementia symptoms with information on the six areas of memory, orientation, judgment and problem solving, community affairs, home and hobbies, and personal care. By integrating the assessments on the six areas, each participant was assigned to healthy (CDR of 0), very mild dementia (CDR of 0.5), mild dementia (CDR of 1), moderate dementia (CDR of 2), and severe dementia (CDR of 3). (2) The Mini-Mental State Examination (MMSE; Folstein et al., 1975) was used as a global measure of cognitive function including orientation, memory, and numeracy skills. (3) We used the Logical Memory II test of the Wechsler Memory Scale-Revised (WMS-R LMII; Wechsler, 1987) to evaluate delayed recall as a measure of episodic memory performance. (4) Finally, the 15-item Geriatric Depression Scale (GDS-15; Sheikh and Yesavage, 1986) was used to assess depressive symptoms. The cutoff point of the GDS-15 was determined as 4/5 (Almeida and Almeida, 1999). Participants with GDS-15 scores of 5 or more were judged depressive and those with scores of 4 or less were judged non-depressive. In the present study, to capture the widest possible range of MCI conditions, participants with a CDR of 0.5 were assigned to the MCI group (DeCarli et al., 2004; Celone et al., 2006; Miller et al., 2008). Those with a CDR of 0 were classified as HCs. Those with a CDR of 1 or greater were excluded from the study because participants with MCI constituted the target population. The 322 participants consisted of 186 individuals with MCI and 136 HCs.

### MRI acquisition

Brain scans for the Osaki-Tajiri and Kurihara projects were acquired on Achieva 1.5T MRI scanners (Philips Medical Systems, Best, Netherlands) by using a sagittal 3D T1-weighted turbo field echo (3D T1-TFE) sequence and an axial 2D FLAIR sequence with eight-channel phased array head coils. The acquisition parameters for the 3D T1-TFE sequence were as follows: repetition time (TR), 9.3 ms; echo time (TE), 4.6 ms; flip angle (FA), 8° for Osaki-Tajiri and 10° for Kurihara; field of view (FOV), 240 mm; and in-plane resolution, 256 × 256 (0.94 × 0.94 mm) with a slice thickness of 1 mm and no intersection gap. Axial FLAIR images were scanned with the following parameters: TR/TE/inversion time (TI), 11000/140/2800 ms for Osaki-Tajiri and 8000/120/2500 ms for Kurihara; FA, 90°; FOV, 230 mm; in-plane resolution, 512 × 512 (0.45 × 0.45 mm); and slice thickness/intersection gap, 5/0.5 mm for Osaki-Tajiri and 6/1 mm for Kurihara.

### Image processing

#### Measurement of cortical thickness

The cerebral cortical thickness of each participant was estimated from the 3D T1-TFE image by using antsCorticalThickness.sh (Tustison et al., 2014), a shell script in the Advanced Normalization Tools (ANTs) software package development version^1^. The image processing workflow is summarized as follows: (1) initial N4 bias correction for alleviating intensity inhomogeneity (Tustison et al., 2010); (2) skull-stripping by using a study-specific template created in advance from 30 participants (20 randomly selected participants with MCI and 10 HCs) with the Symmetric Group Normalization framework (SyGN; Avants et al., 2010) implemented in antsMultivariateTemplateConstruction2.sh; (3) six-tissue segmentation with Atropos (Avants et al., 2011); (4) cortical thickness estimation with DiReCT (Das et al., 2009); and (5) nonlinear spatial registration to the template with SyN (Avants et al., 2008). Manual corrections were added to the results of the skull-stripping as needed by using ITK-SNAP version 3.0.0^2^ (Yushkevich et al., 2006) and a Cintiq 13HD tablet (Wacom Co., Ltd., Kazo, Japan). The six-tissue priors for the study-specific brain template were generated using 3D T1-weighted images from the Open Access Series of Imaging Studies (OASIS) database^3^ (Marcus et al., 2007) and the corresponding expertly and manually segmented labels. These data had been used for the MICCAI 2012 Grand Challenge and the Workshop on Multi-Atlas Labeling^4^, and released under the Creative Commons Attribution-NonCommercial (CC BY-NC) license. The labels were provided by Neuromorphometrics, Inc. (Somerville, MA, USA)^5^ under an academic subscription. The thickness map for each individual was transformed to the template space, using Gaussian interpolation with a sigma of two voxels.

#### Measurement of white matter hyperintensity

White matter hyperintensity segmentation was performed by using the Lesion Segmentation Toolbox version 1.2.3^6^ (Schmidt et al., 2012). The initial threshold for the lesion growth algorithm was set at 0.30 in the current study. The optimal initial threshold value of 0.30 was determined by visually comparing the resultant WMH probability lesion maps derived by using various thresholds between 0.05 and 1.0. Furthermore, gray matter and white matter were empirically chosen as lesion belief maps, whereas only gray matter was set to the lesion belief map by default. Any infratentorial WMH in each image was excluded from the resultant WMH probability map of the whole brain by applying a supratentorial parenchymal mask generated in the processing of cortical thickness (i.e., Atropos six-tissue segmentation). Each WMH probability map was visually inspected and nonlinearly transformed to the study-specific template space by applying the warp estimated in registering the 3D T1-TFE image to the template. The spatially normalized WMH probability maps were then smoothed by using a Gaussian kernel with a sigma of two voxels.

### Statistical analysis

Statistical analyses of demographic data and differences between the MCI and HC groups and between participants with and without depression in the MCI group were performed by using R version 3.1.0^7^ (R Core Team, 2014). Continuous variables were tested for normality by using the Shapiro-Wilk test. Means for age, years of education, neuropsychological examination scores, and WMH ratio (WMHr; the cerebral WMH as a percentage of the total intracranial volume estimated with VBM8) were compared between the MCI and HC groups and between participants with and without depression in the MCI group by the Wilcoxon rank sum test owing to their non-normal distributions. Frequency distributions for gender between the MCI and HC groups and between participants with and without depression in the MCI group were compared by using chi-square tests.

Statistics for voxel-wise image analyses were performed with a voxel-wise general linear model implemented in the FMRIB Software Library (FSL), version 5.0.6^8^ (Jenkinson et al., 2012). Differences in cerebral cortical thickness and WMH probability between MCI and HC participants were tested by using independent sample *t*-tests. Regional associations between cerebral cortical thickness or WMH probability and WMS-R LMII scores within MCI participants were assessed by linear regression analyses, setting cortical thickness or WMH probability as the independent variable and a WMS-R LMII score as the dependent variable. Differences in cortical thickness and WMH probability between MCI participants with GDS-15 scores of 4 or less and those with scores of 5 or more were compared by independent sample *t*-tests. Associations between cerebral cortical thickness and WMHr were also assessed by linear regression analyses, setting cortical thickness as the independent variable and WMHr as the dependent variable. All voxel-wise statistical analyses including *t*-tests and linear regression analyses used age, gender, and years of education as covariates.

Permutation-based nonparametric testing was carried out by the “randomize” function in FSL with 5000 permutations (Nichols and Holmes, 2002), using gray and white matter masks for analyses of cortical thickness and WMH probability, respectively. The gray matter and white matter masks were generated by binarizing the corresponding prior probability maps of the template with the lowest probability threshold of 0.5. By using threshold-free cluster enhancement (TFCE; Smith and Nichols, 2009), the results of all statistics were thresholded at *p* < 0.05, family-wise error (FWE)-corrected for multiple comparisons. Threshold-free cluster enhancement was used to avoid arbitrarily setting the smoothing kernel size and using an arbitrary cluster-forming threshold.

## Results

### Demographic and clinical data

A summary of the demographic characteristics and neuropsychological examination scores of the MCI and HC groups is shown in Table 1. The difference in GDS score between the two groups was not statistically significant, whereas the MCI group showed significantly lower MMSE and WMS-R LMII scores and higher WMHr than did the HC group (Wilcoxon rank sum test; *p* < 0.05). Table 2 shows the differences between MCI participants with a GDS score of 4 or less (a non-depressed state) and those with a score of 5 or more (a depressed state). The WMHr of depressed MCI participants was significantly higher than that of non-depressed MCI participants. There were no other significant differences between the two groups.

**Table 2:** **Demographic differences between MCI participants with GDS-15 ≤4 and ≥5**.

	MCI with GDS ≤4	MCI with GDS ≥5	*p*-value
Number	105	81	
Age, y	80 (76–82)	80 (77–83)	0.13
Gender, M/F	45/60	39/42	0.57
Education, y	9 (8–10)	8 (8–9)	0.08
MMSE	23 (21–26)	23 (21–25)	0.77
WMS-R LMII	4 (1–10)	4 (1–8)	0.44
WMHr, %	1.29 (0.75–2.88)*^a^*	2.35 (1.26–3.19)*^a^*	0.006

*Data are presented as numbers or medians (interquartile ranges). Abbreviations: F = female; GDS-15 = 15-item Geriatric Depression Scale; M = male; MCI = mild cognitive impairment; WMHr = cerebral white matter hyperintensity volume as a percentage of the total intracranial volume; WMS-R LMII = Wechsler Memory Scale-Revised Logical Memory II. ^a^*p* < 0.01*.

### Differences in cortical thickness and WMH probability between MCI and HC

**Figure 2:**
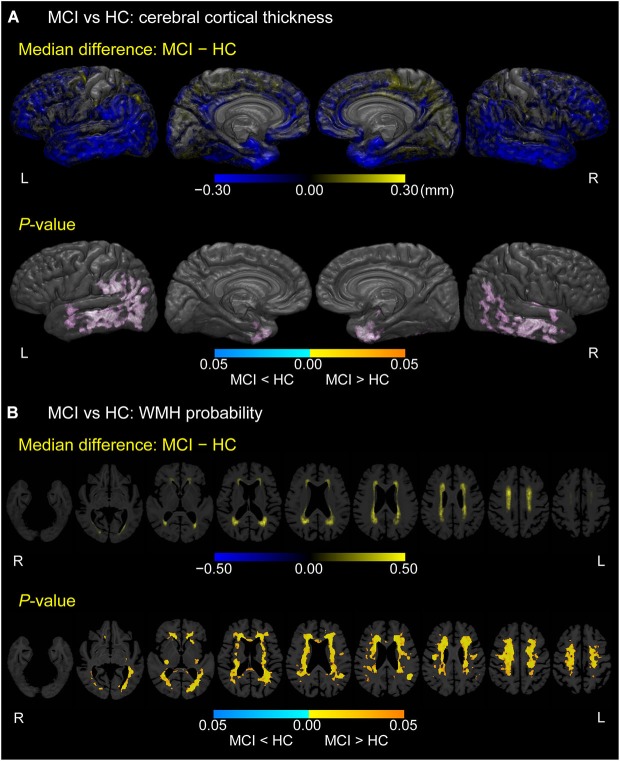
**Difference between MCI and HC groups in cerebral cortical thickness and WMH probability. (A)** Cortical maps of median differences in cortical thickness (MCI − HC; in millimeters) and family-wise error (FWE)-corrected *p*-values between participants with MCI and HCs. **(B)** White matter maps of median differences in WMH probability (MCI − HC) and FWE-corrected *p*-values between participants with MCI and HCs. These results are overlaid on the skull-stripped study-specific template, adjusted for effects of age, gender, and years of education.

As displayed in Figure 2A, MCI participants exhibited thinner cortices than HCs mainly in the bilateral temporal pole, fusiform gyrus, anterior parahippocampal gyrus (anterior part of the entorhinal cortex), middle and inferior temporal gyrus, inferior parietal gyrus, and left supramarginal gyrus (*p* < 0.05, FWE-corrected). In Figure 2B, MCI participants also showed higher WMH probabilities than HCs in bilateral cerebral white matter, especially in the corona radiata, semioval center, and regions anterior to the genu of the corpus callosum and around the trigone and anterior horn of the lateral ventricle (*p* < 0.05, FWE-corrected).

### Association between episodic memory score and cortical thickness or WMH probability in MCI

**Figure 3:**
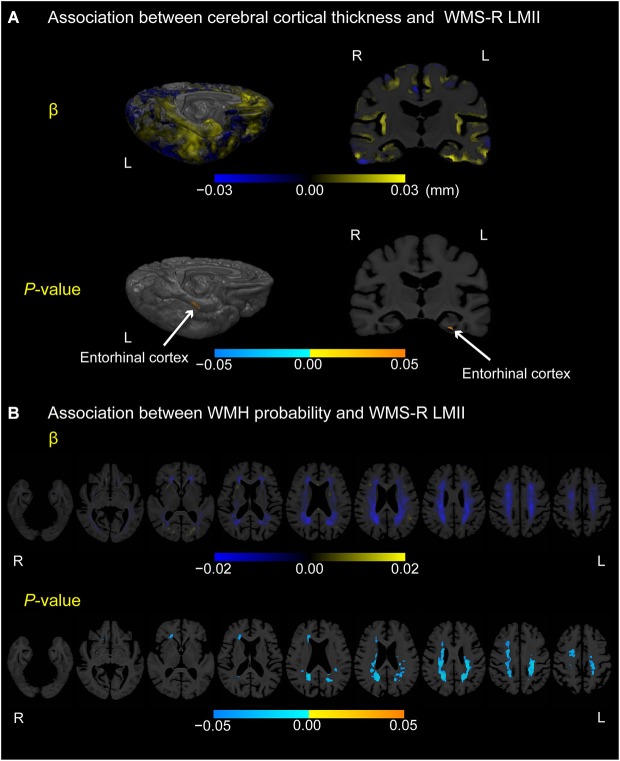
**Association between cerebral cortical thickness and WMH probability or WMS-R LMII score in participants with MCI. (A)** Cortical maps of regression coefficients (unstandardized beta values) and FWE-corrected *p*-values. The regression coefficients represent the estimated contribution to cerebral cortical thickness as millimeters per unit increase in WMS-R LMII score. **(B)** White matter maps of regression coefficients (unstandardized beta values) and FWE-corrected *p*-values. The regression coefficients represent the estimated contribution to WMH probability per unit increase in the WMS-R LMII score. These results are overlaid on the skull-stripped study-specific template, adjusted for effects of age, gender, and years of education. White arrows in **(A)** point to the left entorhinal cortex.

Figure 3 displays locations in the cerebral cortex or white matter where cerebral cortical thickness (A) or WMH probability (B), respectively, was associated with the episodic memory score (i.e., WMS-R LMII score) in participants with MCI (*p* < 0.05, FWE-corrected). Only the most posterior part of the left entorhinal cortex exhibited a positive correlation between cortical thickness and the episodic memory score. In contrast, negative correlations between WMH probability and episodic memory score were detected mainly in the bilateral posterior periventricular regions and near the right anterior horn of the lateral ventricle.

### Differences in cortical thickness and WMH probability between depressed and non-depressed MCI participants

**Figure 4:**
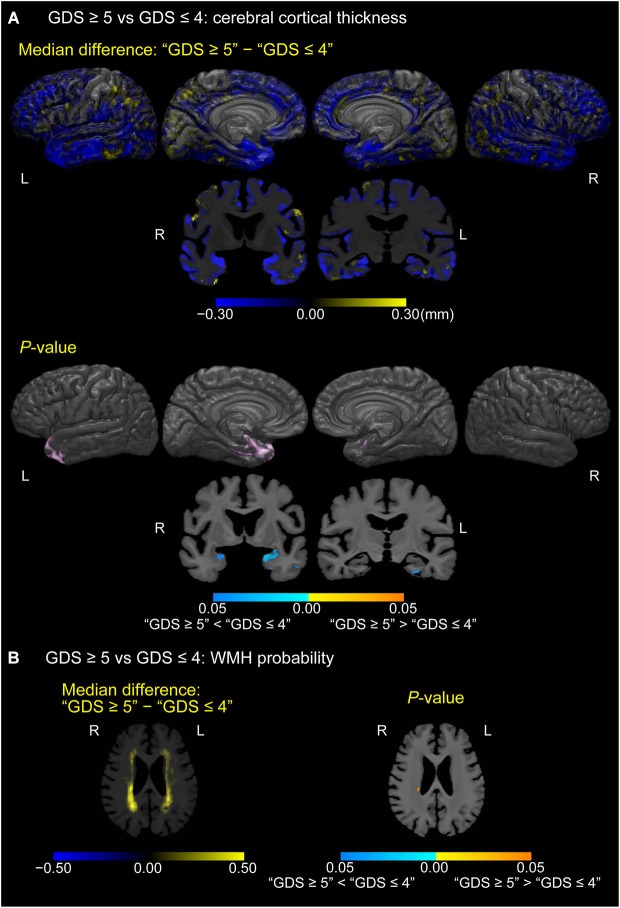
**Differences in cerebral cortical thickness and WMH probability between depressed and non-depressed MCI participants. (A)** Cortical maps of median cortical thickness (MCI with GDS ≥5 − MCI with GDS ≤4; in millimeters) and family-wise error (FWE)-corrected *p*-values between MCI participants with “GDS ≥5” and “GDS ≤4.” **(B)** White matter maps of median differences in WMH probability (“GDS ≥5” − “GDS ≤4”) and FWE-corrected *p*-values between MCI participants with “GDS ≥5” and “GDS ≤4.” These results are overlaid on the skull-stripped study-specific template, adjusted for effects of age, gender, and years of education.

Figure 4 shows cortical or white matter regions where differences in cortical thickness (A) or WMH probability (B), respectively, were observed between depressed MCI participants (GDS score of 5 or more) and non-depressed MCI participants (GDS score of 4 or less; *p* < 0.05, FWE-corrected). Thinner cortical regions were seen in the temporal pole, left parahippocampal gyrus, and uncus of the left hemisphere in the depressed group. Moreover, coronal sections also showed the gyrus adjacent to amygdala to be thinner in depressed MCI participants bilaterally. In contrast, increased WMH probability was seen only in a small region of the right periventricular white matter in depressed MCI participants.

### Associations between cortical thickness and WMH ratio in MCI

**Figure 5:**
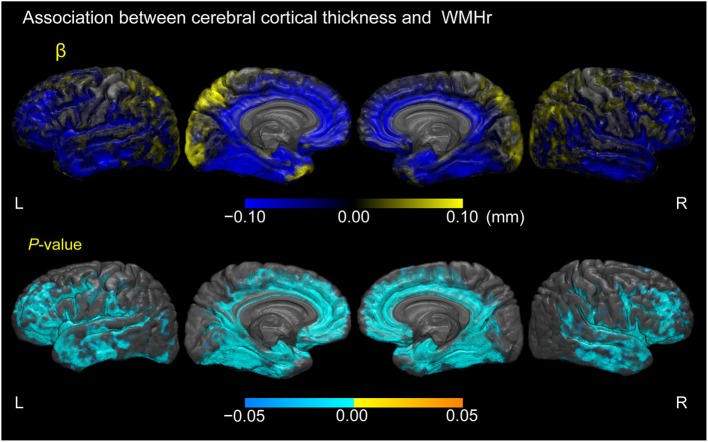
**Association between cerebral cortical thickness and white matter hyperintensity volume as a percentage of the total intracranial volume in participants with MCI**. Cortical maps of regression coefficients (unstandardized beta values) and FWE-corrected *p*-values. The regression coefficients represent the estimated contribution to cerebral cortical thickness as millimeters per unit increase in WMH volume as a percentage of total intracranial volume. These results are overlaid on the skull-stripped study-specific template, adjusted for effects of age, gender, and years of education.

Figure 5 displays cortical regions where a linear regression model estimated associations between cortical thickness and WMHr, adjusted for age, gender, and years of education in participants with MCI (*p* < 0.05, FWE-corrected). Negative correlations between cortical thickness and WMHr were seen broadly in the lateral, medial frontal, and temporal lobes, including the gyrus adjacent to the amygdala and entorhinal cortex in the bilateral hemispheres.

## Discussion

We have explored cortical thickness and WMH volume in connection with MCI, as well as associations among episodic memory, depression, cortical thickness, and WMH in the brain MR scans of participants with MCI (median age, ~80 years old). In participants drawn from two community-based cohort studies in northern Japan, we used automated quantitative procedures to clarify these associations and spatial distributions of cortical thinning as well as increases in WMH volume simultaneously. We obtained four main findings. First, MCI patients have thinner cortices and more WMHs in specific regions of the cerebrum than do HCs. Second, poor episodic memory is associated with reduced cortical thickness in the left entorhinal cortex and increased WMHs in specific locations of the cerebrum. Third, a depressive state is associated with reduced cortical thickness in the left temporal pole, left entorhinal cortex, and gyrus adjacent to the amygdala bilaterally, as well as with a higher WMH volume as a percentage of the total intracranial volume. Fourth, a larger WMH volume is associated with thinner cortices in the lateral, medial frontal, and temporal lobes. These findings indicate that both the cerebral cortex and white matter are altered in MCI patients as changes occur in episodic memory and depressive state.

### Differences in cerebral cortical thickness and WMH between MCI and healthy aging

In this study, at the stage of MCI or mild dementia (classified as CDR 0.5), thinning of the cerebral cortex occurred mainly in the medial and lateral portions of the temporal lobe and the inferior parietal lobe. This finding is in accord with the results of other studies (Singh et al., 2006; Fennema-Notestine et al., 2009). However, the middle and posterior portions of the entorhinal cortex did not exhibit significant differences between MCI participants and HCs in the present study. One possible reason is that MCI participants included not only those with amnestic MCI but also some with non-amnestic MCI, given that a global CDR score of 0.5 was sufficient to classify a participant as having MCI. Hence, the etiologies of MCI could include vascular dementia, depression, frontotemporal dementia, dementia with Lewy bodies, and other dementing disorders as well as AD (Petersen and Morris, 2005).

White matter hyperintensities were more prominent for MCI participants than HCs, particularly in the corona radiata, semioval center, and near the genu of the corpus callosum and anterior horn of the lateral ventricle in the bilateral hemispheres. These regions are distributed along the lateral and medial cholinergic pathways that have been elucidated with immunohistochemical procedures in autopsied brains (Selden et al., 1998). Accordingly, our findings lend support to the previous finding that the severity of WMHs in the cholinergic pathway, as measured by a semi-quantitative visual rating scale, is correlated with cognitive status (Bocti et al., 2005). Our findings are consistent with the hypothesis of the authors that WMHs disrupt the cholinergic pathway and contribute to deterioration of cognitive function.

### Associations among episodic memory, cortical thickness, and WMH

We confirmed a relationship between episodic memory and cerebral cortical thickness. Our finding suggests that the left entorhinal cortex is involved in episodic memory. Similar findings have been observed by others (Di Paola et al., 2007; Sarazin et al., 2010) in individuals with AD by using voxel-based morphometry (VBM). Hippocampal atrophy is also reportedly associated with poor episodic memory in studies using VBM (Chetelat et al., 2003; Leube et al., 2008; Sarazin et al., 2010) although we did not assess the hippocampal volume using VBM in the present study. A recent study has reported that, compared with VBM, the cortical thickness measure is better at detecting pathological changes in the cerebral cortex (Diaz-de-Grenu et al., 2014). Given that the entorhinal cortex is thin and located in a relatively small area, smoothing with a large kernel size—a method commonly adopted in VBM procedures—might induce contamination from voxels in adjacent areas and reduce the sensitivity to relatively slight atrophic changes of this structure in MCI patients.

We observed that episodic memory was negatively correlated with WMH volume in the region near the right anterior horn of the lateral ventricle as well as the bilateral posterior periventricular white matter, specifically in the parietal-occipital area. These regions contain the superior longitudinal fasciculus including the arcuate fasciculus, as well as the posterior and superior thalamic radiations (Mori et al., 2005). Our findings suggest that WMHs directly impair episodic memory by disrupting the thalamocortical connections as well as the connections between the parietal and temporal cortices and prefrontal cortex. However, our findings are not in line with those of Smith et al. (2011), who showed an association between episodic memory and WMH volume in the temporal-occipital and parietal periventricular white matter and internal capsule (anterior limb). The discrepancy may be partly explained by differences in subject population depending on whether the selection criteria included individuals with major vascular risk factors.

### Associations among depressive state, cortical thickness, and WMH

In the present study, the depressive state in MCI participants was associated with reduced thickness in the left temporal pole, left entorhinal cortex, and gyrus adjacent to the amygdala bilaterally. These results partially concur with the results of some who reported a relationship between late-onset depression and volume reduction in the amygdala (Egger et al., 2008; Burke et al., 2011) or entorhinal cortex (Gerritsen et al., 2011). However, others have reported that acceleration of hippocampal atrophy was associated with late-onset depression in a longitudinal study (den Heijer et al., 2011). Although the reason for these differences is unclear, they may be attributable to differences in the method of image processing. Furthermore, they may also be attributed to differences in the subject population, because only participants with MCI were analyzed to assess the relationship between brain atrophy and depressive state in the present study.

The total WMH volumes in depressed MCI individuals were higher than those in non-depressed participants, although only a small suprathreshold cluster was observed in the right periventricular white matter. Our finding is congruent with a meta-analysis that has shown that individuals with late-life depression have more frequent and severe WMHs than do HCs (Herrmann et al., 2008). Some research groups have reported that greater WMH volume in the superior longitudinal fasciculus, uncinate fasciculus, or cingulum bundle is relevant to late-life depression (Sheline et al., 2008; Dalby et al., 2010; Taylor et al., 2013). The disparity between the results of these groups and our findings may be related to the fact that we simply assigned MCI participants to two groups based on GDS-15 scores, instead of using operational diagnostic criteria such as the Diagnostic and Statistical Manual of Mental Disorders, Fourth Edition, Text Revision (DSM-IV-TR; American Psychiatric Association, 2000) to obtain a diagnosis for depression with higher reliability.

### Relationship between cortical thickness and WMH burden

We found that larger WMH volumes were associated with thinner cortices in the lateral, medial frontal, and temporal lobes. This finding is roughly in keeping with that reported by Ye et al. (2014) despite methodological differences in measuring cortical thickness and WMH. The result may indicate that WMHs contribute to cerebral cortical thinning by disrupting specific fiber tracts additively or synergistically with pathophysiological processes in AD (Bloom, 2014). We observed that WMHs were involved in cerebral cortical thinning across broad areas of the cerebrum including the frontal cortex and posterior cingulate. According to a recent study, cortical thickness in the prefrontal cortex and that in the posterior cingulate are associated with executive function in MCI patients (Chang et al., 2010). Although we did not assess executive function in the present study, our results lead us to surmise that WMHs contribute to poor executive function by causing cerebral cortical thinning in these regions.

### Influence of cerebral cortical thinning and WMH burden on MCI and depression

We cannot determine whether Wallerian or retrograde degeneration is involved in the relationship between cortical thinning and increased WMH volume in MCI and late-life depression. However, Brickman et al. (2014) reported in their longitudinal study that higher baseline parietal WMH volume, increasing parietal WMH volume, smaller baseline hippocampal volume, and atrophic change in hippocampal volume independently predicted progression to AD from a non-demented state. Measures of cortical thickness did not predict disease progression. Moreover, in a longitudinal study, Duering et al. (2012) reported marked thinning of the cerebral cortex in regions that showed a high probability of connectivity with incident subcortical infarcts. Thus, cortical thinning in a specific area might occur secondarily after incident subcortical WMHs connected with the cortical area. Therefore, WMH volume may have greater influence on MCI than cortical thinning.

One possible interpretation of the associations among cortical thinning in the medial temporal lobe, increased WMH volume, and depression is that some patients have depression as an early symptom associated with a neurodegenerative process that leads to dementia, as discussed by Lebedeva et al. (2014), whereas WMHs may predispose an individual to or precipitate depression as well as perpetuate pre-existing depression (Alexopoulos et al., 1997).

### Limitations of this study

This study has 10 principal limitations. First, we could not assess pathological microstructural alterations in cerebral white matter by diffusion tensor imaging (DTI), which has the potential to evaluate changes in white matter tracts that appear normal with T2-weighted images or FLAIR (Shimony et al., 2009; Papma et al., 2014) as well as sites of pathological changes in cerebral white matter tracts. Second, we used 2D FLAIR instead of 3D FLAIR owing to equipment limitations. The segmentation results were visually inspected with care because the automated WMH segmentation process adopted here was designed mainly for 3D FLAIR (Schmidt et al., 2012). However, 3D FLAIR images should be obtained and assessed in a future study with a newer automated WMH segmentation program that is optimized for 3D FLAIR and is now publicly available (Ithapu et al., 2014). Third, because participants with a CDR of 0.5 were classified into the MCI group as noted above, the etiologies for this group could include AD, vascular dementia, depression, frontotemporal dementia, dementia with Lewy bodies, and mixed dementia. Therefore, to test whether AD, for example, is the primary etiology for such participants given its primary role in causing dementia (Querfurth and LaFerla, 2010), participants in future studies could be additionally tested for amyloid beta peptide and tau in cerebrospinal fluid or for amyloid and fluorodeoxyglucose by positron emission tomography (Albert et al., 2011). Fourth, the method of cortical thickness estimation adopted here could not assess the volume of the amygdala or hippocampus. We found no significant differences in cortical thickness between MCI participants and HCs or between depressed and non-depressed MCI participants, unlike previous reports that have frequently reported hippocampal volume loss (Ballmaier et al., 2008; Steffens et al., 2011; den Heijer et al., 2011). Therefore, subcortical structures might be assessed more effectively in the future with surface shape analysis (Ballmaier et al., 2008; Zhao et al., 2008; Costafreda et al., 2011; Devanand et al., 2012). Fifth, we used a GDS-15 cut-off value to determine the depressive state of each participant. This could have resulted in overdiagnosis of depression. In future studies, depression should be diagnosed according to the criteria of DSM-IV-TR or the 10th revision of the International Classification of Diseases (ICD-10; World Health Organization, 1992) and assessed for severity by the Hamilton or Montgomery–Asberg Depression Rating Scale (Hamilton, 1960; Montgomery and Asberg, 1979). Additional information about patient history, for example, of attempts or current desire to commit suicide, might also yield interesting results from voxel-wise analyses of cerebral cortical thickness or WMH probability. The sixth limitation is the cross-sectional nature of this study. Clarifying the dynamic associations between increasing WMH volume or cortical thinning and changes in cognitive functions or affective states, for instance, will require intra-individual serial MRI and repeated neuropsychological assessments for a specified period. The seventh limitation is the lack of accounting for cognitive effects of psychoactive agents. For example, participants with depression who take antidepressants could have an improved GDS-15 score and might therefore be assigned to healthy elderly controls despite having some atrophy in specific brain areas due to the disease. The eighth limitation is that we did not control for the effects of vascular risk factors (e.g, hypertension, history of cardiovascular disease, diabetes mellitus, cigarette smoking) associated with increased WMH volume (Jeerakathil et al., 2004; Tiehuis et al., 2008). The ninth limitation is the selection bias owing to the different criteria of the two projects. The Osaki-Kurihara project recruited participants aged over 65, whereas the Kurihara project recruited participants aged over 75. The prevalence of WMHs could be higher in the participants of the Kurihara than those of the Osaki-Tajiri. The tenth limitation is the heterogeneity of the slice thickness and intersection gap of 2D FLAIR sequence between the two projects. Although we did not examine any difference in WMH volume between the participants of the two projects, the heterogeneity might affect the measurement of WMH volume.

## Conclusion

In this study, we simultaneously revealed an association between reduced cortical thickness and increased WMH volume in MCI, as well as associations among episodic memory, depressive state, cortical thickness, and WMHs and their spatial distributions in a single population. We found that MCI, poor memory performance, and depressive state involve both cortical thinning and WMH expansion in specific regions, and that increased total WMH volume is closely associated with cortical thinning. Our results verify the importance of clarifying specific pathophysiologic processes by examining combinations of cerebrovascular lesions, AD, and other dementing disorders in future studies (Kling et al., 2013). In addition, they support the view that cerebrovascular lesions could contribute to the pathogenesis of late-life depression (Alexopoulos et al., 1997). Further longitudinal studies of cortical thinning and white matter disruption in elderly individuals with MCI are needed to elucidate the pathophysiological processes of dementia and late-life depression in greater detail.

## Author and contributors

Conception and design of the present study: Motonobu Fujishima. Acquisition of data: Kei Nakamura, Masahiro Nakatsuka, and Kenichi Meguro. Analysis of data: Motonobu Fujishima and Norihide Maikusa. Interpretation of data: Motonobu Fujishima and Hiroshi Matsuda. Preparation of manuscript: Motonobu Fujishima and Hiroshi Matsuda. Supervision: Hiroshi Matsuda.

## Conflict of interest statement

The authors declare that the research was conducted in the absence of any commercial or financial relationships that could be construed as a potential conflict of interest.

## Footnotes

^1^http://stnava.github.io/ANTs

^2^http://www.itksnap.org

^3^http://www.oasis-brains.org

^4^https://masi.vuse.vanderbilt.edu/workshop2012

^5^http://www.neuromorphometrics.com

^6^http://www.applied-statistics.de/lst.html

^7^http://www.r-project.org/

^8^http://fsl.fmrib.ox.ac.uk/fsl/
